# Neutropénie fébrile chimio-induite dans une unité d’oncologie pédiatrique tunisienne

**DOI:** 10.11604/pamj.2022.42.34.28176

**Published:** 2022-05-12

**Authors:** Faten Fedhila, Sarra Ben Ahmed, Elhem Jbebli, Fatma Mezghani, Samir Haddad, Samar Rhayem, Monia Khemiri

**Affiliations:** 1 Service de Médecine Infantile A, Hôpital d’enfants Béchir Hamza, Tunis, Tunisie

**Keywords:** Neutropénie fébrile, enfant, tumeurs, microbiologie, chimiothérapie, Febrile neutropenia, pediatric, tumors, microbiology, chemotherapy

## Abstract

La localisation mammaire isolée d´un lymphome de Hodgkin est rare. Elle peut prêter à confusion avec les autres néoplasies et affections inflammatoires mammaires. Nous rapportons le cas d´une patiente âgée de 18 ans traitée pour lymphome de Hodgkin à cellularité mixte, qui s´est présenté 6 mois après la rémission complète pour un nodule mammaire inflammatoire gauche. Le bilan anatomopathologique initial a été en faveur d´un abcès mammaire. Les investigations face à l´évolution défavorable malgré une antibiothérapie adaptée ont été en faveur d´une rechute du LH avec une localisation mammaire isolée. La patiente est actuellement sous 4^e^ ligne thérapeutique (polychimiothérapie). Les lymphomes Hodgkiniens mammaires sont connus par leur pronostic défavorable. Les progrès thérapeutiques (thérapie ciblée) peuvent améliorer le devenir des patientes.

## Abstract (en)

Chemotherapy-induced neutropenia (FN) is the most common infectious complication in pediatric oncology. To our knowledge, no pediatric research has been published in Tunisia. The purpose of our study was to describe the features of FN among Tunisian children and to investigate factors correlated with FN. We conducted a prospective study of children with chemotherapy-induced FN at the Department of Pediatric Medicine A of the Tunis Children´s Hospital from July 2019 to December 2019. We recorded 50 episodes of FN in 32 patients whose mean age was 5.3 years (3 months-16 years). We included 26 patients with solid tumors (81%) and six patients with hemopathies (18.7%). The mean time between last treatment and fever onset was 10.67 days. Bacteriological investigation was contributory in 18% of cases and mainly showed gram positive cocci. Therapeutic protocol including 1^st^ line empirical antibiotic therapy (3^rd^ generation cephalosporin with aminoglycoside) was effective in 62% of cases. Mortality rate of patients with FN was 2%. The statistical study did not reveal any factor of correlation with late-onset neutropenia. In conclusion, our results are consistent with literature data on bacteriological documentation and mortality. Our 1^st^ line treatment option based on 3^rd^ generation cephalosporin associated with aminoglycoside was effective in 2/3 of the cases. In the future, oral antibiotics may be considered in patients at low risk for infection.

## Introduction

La neutropénie fébrile (NF) constitue une urgence médicale en oncologie pédiatrique. Il s´agit de la principale complication infectieuse chez les enfants traités par chimiothérapie. Sa gravité dépend de la profondeur, de la durée de l´immunosuppression, des co-morbidités associées et de la qualité de la prise en charge [1,2]. Cette situation nécessite l´administration d´une antibiothérapie parentérale de manière empirique selon des recommandations communément admises et adaptées à l´écologie bactérienne de la structure hospitalière [3,4]. Ces recommandations, appliquées depuis les années 80 [5], ont permis de réduire le taux de mortalité, estimé à 30 % dans les années 1970, à 0,25-4% dans les années 2000 en pédiatrie [3]. A notre connaissance, aucune étude pédiatrique Tunisienne portant sur les NF chimio-induites n´a été publiée à ce jour. A ce propos, nous avons conduit une étude prospective portant sur les NF chimio-induites dans une population pédiatrique. Les objectifs de cette étude étaient de décrire les caractéristiques socio-démographiques, cliniques, microbiologiques et évolutives des NF chimio-induites dans une unité d´oncologie pédiatrique et de rechercher une corrélation entre les facteurs cliniques, microbiologiques, biologiques et la durée de la neutropénie.

## Méthodes

**Type et lieu de l´étude:** il s´agit d´une étude prospective, descriptive longitudinale conduite dans l´unité d´oncologie pédiatrique du service de Médecine Infantile A de l´hôpital d´enfants Béchir Hamza de Tunis sur une période de 6 mois [Juillet 2019- Décembre 2019].

**Population:** nous avons inclus les enfants âgés de moins de 18 ans suivis pour une pathologie tumorale (hémopathie ou tumeur solide) traités par chimiothérapie conventionnelle, et présentant une NF répondant aux critères de la Société Américaine des Maladies Infectieuses (IDSA). Un consentement éclairé a été pris avant le recueil des données auprès de leurs tuteurs. L´étude a été approuvée par le comité d´éthique de l´Hôpital d´Enfants de Tunis. La NF a été définie selon l´IDSA [6] par l´association d´un taux de Polynucléaires neutrophiles (PNN) < 500 éléments /mm^3^ ou compris entre 500 et 1000 éléments/ mm^3^ susceptibles de devenir < 500 éléments/mm^3^ dans les 24h et d´une fièvre ≥ 38,5° une fois ou bien >38° à deux reprises à une heure d´intervalle. Les NF ont été classées selon l´IDSA [6] en 3 catégories: a) une fièvre microbiologiquement documentée (FMD) définie par la mise en évidence d´une bactériémie ou d´une culture positive d´un prélèvement microbiologique au niveau d´un foyer infectieux. b) Une fièvre cliniquement documentée (FCD) définie par la présence d´un foyer infectieux clinique sans documentation microbiologique de certitude. C) Une fièvre d´origine indéterminée (FOI) correspond à la présence d´une fièvre récente isolée, en l´absence de foyer clinique après un examen clinique minutieux et en l´absence d´isolement d´un pathogène à la microbiologie.

**Figure 1:**
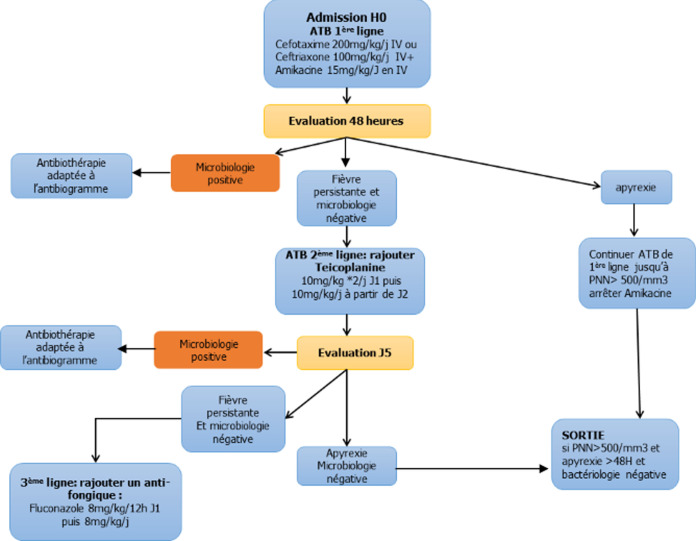
algorithme décisionnel de l´antibio thérapie probabiliste

**Data collection:** pour chaque épisode de NF, différentes données ont été recueillies de façon prospective à l´aide d´une fiche préalablement établie: données socio-démographiques, cliniques, para-cliniques, thérapeutiques et évolutives. Tous les patients ont eu un examen clinique complet, un bilan biologique incluant un hémogramme, une C-Reactive protein (CRP), un ionogramme sanguin, des hémocultures, un examen cytobactériologique des urines, un prélèvement local d´une éventuelle porte cutanée, des coprocultures en cas de diarrhée et une Radio du thorax en cas de signes respiratoires. Une échographie abdominale a été pratiquée en cas de diarrhée accompagnée de douleurs abdominales et de vomissements à la recherche d´un aspect évocateur de typhlite (entérocolite neutropénique). Les patients ont été traités selon une stratégie qui prend en compte l´évolution clinique et biologique selon un algorithme précis (Figure 1).

**Tableau 1:** répartition des anomalies retrouvées à l´examen clinique

	Signes cliniques		N=50	Pourcentage(%)
**Cutanéo-muqueux**	Mucite	grade 1	5	32
		grade 2	5	
		grade 3	6	
	Cellulite	Cellulite orbitaire	1	4
		Cellulite de la cuisse droite	1	
	Marbrures		1	2
	Aphtes buccaux		1	2
	Conjonctivite		1	2
	Issue de pus par une plaie		1	2
**ORL**	Angine		2	4
**Cardiovasculaire**	Etat de choc hypovolémique		1	2
**Pleuro-pulmonaire**	Râles crépitants à l´auscultation		1	2
**Abdominal**	Ballonnement abdominal avec sensibilité abdominale		1	2

**Analyse statistique:** les données ont été saisies et analysées à l´aide du logiciel Statistical Package for the Social Sciences (SPSS) version 20. Pour les variables quantitatives, nous avons calculé des moyennes, des écarts-type, avec détermination des valeurs extrêmes, et des médianes. Pour les variables qualitatives, nous avons déterminé les pourcentages relatifs à chaque catégorie. Afin d´identifier les facteurs prédictifs liés à la durée de la NF, nous avons conduit une analyse univariée (facteur par facteur) par le test du Log Rank. Le seuil de signification a été fixé à 0,05.

## Résultats

**Figure 2:**
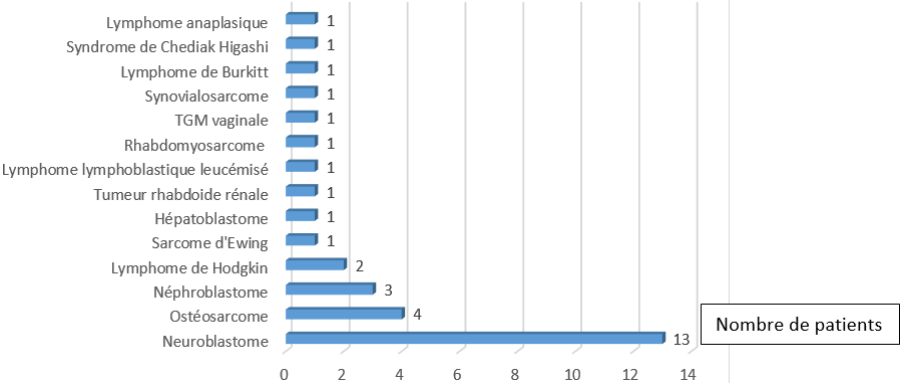
répartition des patients en fonction du type de pathologie

Nous avons colligé 50 épisodes de NF, survenus chez 32 patients dont l´âge moyen était de 5,3 ans±4,29 ans (extrêmes: 3 mois-16 ans). Nous avons colligé 81,25% de tumeurs solides (n=26) avec une prédominance du neuroblastome dans 40,6% des cas suivis par les sarcomes osseux (15,6%) (Figure 2). Le délai moyen entre le premier jour de la dernière cure et la fièvre était de 10,67 jours (1-31 jours). La durée moyenne de la fièvre avant l´hospitalisation était de 8,4 heures (extrêmes: 1/2-48 heures). Des signes de mauvaise tolérance ont été rapportés dans 4% des cas (n=2). Les signes fonctionnels associés à la fièvre rapportés dans 19 épisodes (38%) étaient dominés par les troubles digestifs dans 15/19 cas (78,9%), suivis par les manifestations respiratoires dans 4 cas (21%). Dans 54% des épisodes de NF, l´examen clinique n´a pas révélé de foyer infectieux (Tableau 1). Sur le plan biologique, le taux moyen des polynucléaires neutrophiles (PNN) était de 176,9/mm^3^(0-500/mm^3^). Une neutropénie profonde, soit PNN<100/mm^3^, était présente dans 22 cas (44%). Le taux moyen de la CRP était de 59,8 mg/l (113-321mg/l).

Les hémocultures périphériques pratiquées chez tous les malades étaient positives dans 7 cas (14%). Les hémocultures centrales sur chambre implantable pratiquées dans trois cas étaient négatives (Tableau 2). Au terme de cette étude, nous avons pu classer les épisodes de NF en trois catégories selon la classification internationale établie par l´IDSA: fièvre d´origine indéterminée (28%), fièvre cliniquement documentée (54%) et fièvre microbiologiquement documentée (18%). Tous les patients ont reçu une antibiothérapie de 1^ère^ ligne par voie intraveineuse associant une céphalosporine de 3^e^ génération (C3G) et un aminoside (Figure 1). Elle a été exclusive dans 31 cas (62%). Devant la persistance de la fièvre et la négativité de l´enquête microbiologique, une antibiothérapie de 2^e^ ligne de type Teicoplanine a été associée à H48 dans 19 cas (38%) et un antifongique a été rajouté en 3^e^ ligne à J5 dans 3 cas (6%). La durée totale du traitement de la NF était de 7,58 jours (3-19 jours). Un traitement par des facteurs de stimulation des colonies de granulocytes (G-CSF) a été prescrit dans 8 cas (16%) après un délai moyen de 3,3 jours (1-6jours). La durée moyenne de la fièvre était de 53,6 ± 47 heures (6-216 heures). L´apyrexie a été obtenue au bout de 48h dans 31 cas (62%). La durée moyenne de la neutropénie était de 7,3 jours± 3,17 jours (3-22j). Un patient suivi pour un ostéosarcome et traité par chimiothérapie myéloablative (Adriamycine, cisplatine, Ifosfamide) est décédé dans un tableau d´état de choc septique soit un taux de mortalité imputé à l´infection de 2%. L´étude statistique a montré qu´il n´y avait pas de corrélation statistiquement significative entre la durée de la neutropénie et le nombre d´épisodes de NF antérieurs (p=1), la pathologie sous jacente (p=1), la mucite (p=0.35), la sévérité de la neutropénie (p=0.065), la valeur de la CRP (p=0.65) et la positivité de l´enquête bactériologique (p=0.875).

**Tableau 2:** répartition des germes en fonction du site d´isolement

	Hémocultures n=50		Coprocultures n=4		Prélèvements cutanés n=2	
	Staphylocoque coagulase négative	3	Salmonella	1	Staphylocoque coagulase négative	1
	Entérocoque	1				
	Klebsiella Pneumoniae BLSE	1				
**Germes isolés**	Alcaligenes Denitrificans	1	Négatives	3	Négatif	1
	Salmonella	1	Négatives	3	Négatif	1
	Négatives	48				

**BLSE** : Bétalactamase à spectre élargi

## Discussion

En pédiatrie, l´incidence de l´aplasie fébrile post-chimiothérapie conventionnelle est de 25 à 40% [1] contre 6 à 8% chez l´adulte [5] du fait d´une altération de l'immunité innée et adaptative, expliquant le risque élevé de morbidité et de mortalité [4]. Dans notre série, les tumeurs solides représentaient plus des ¾ des pathologies tumorales (81,25%) avec une prédominance des neuroblastomes suivis par les sarcomes osseux. Les sarcomes osseux sont en fait les tumeurs les plus pourvoyeuses d´épisodes de NF, comme en attestent les séries de Hodgson-Viden *et al*. Al Omar *et al*. et Mian *et al*. où ils sont retrouvés respectivement dans 17,32%, 10,09% et 25% des cas [6,7]. Les neuroblastomes détiennent la deuxième place en termes de fréquence dans les séries suscitées. Ceci est expliqué par l´utilisation de chimiothérapies aplasiantes telles que la Doxorubicine, l´Ifosfamide, le Cyclophosphamide, l´Etoposide et à la durée prolongée de la chimiothérapie dans certains protocoles. D´après l´étude de Le Carrer *et al*. [8], la moyenne de survenue de la NF après le premier jour de la cure de chimiothérapie est de 13 jours versus 10,67 jours dans notre étude. Dans les séries d´enfants traités pour des hémopathies, ce délai est plus prolongé, *et al*. comme rapporté par Salhi *et al*. [9]. La fièvre demeure le signe le plus constant de la NF et parfois même l´unique. La présentation clinique chez l´enfant neutropénique est en effet le plus souvent pauvre ce qui était le cas dans notre étude. La mucite prédomine dans notre série ce qui concorde avec les différentes séries : 69% dans l´étude de Le Carrer [8] et 62% dans celle d´Al Omar [7]. Ainsi, des symptômes et des signes cliniques absents ou frustes chez des patients pauci-symptomatiques ne doivent en aucun cas rassurer le clinicien, et retarder la mise en route de l´antibiothérapie.

Dans notre série, la documentation microbiologique a été rapportée dans 18% des épisodes de NF versus 10 à 24 % dans la littérature [3]. Le profil bactérien dans notre série était représenté majoritairement par des CGP suivis par les BGN, se rapprochant des données de la littérature (70% de CGP et 30% de BGN) avec une prédominance du *Staphylocoque* coagulase négative (50 à 70%) et des Streptocoques (5 à 15%). L´émergence des CGP comme c´est le cas dans notre série, peut s´expliquer par différents facteurs: l´utilisation croissante des cathéters centraux, les mucites chimio-induites favorisant surtout les infections à Streptocoque, l´utilisation prophylactique des quinolones, qui exercent une pression de sélection sur certains CGP [10]. Pour les BGN, l´*Escherichia coli* domine nettement le profil contre moins de 5% de *Pseudomonas aeruginosa* [11]. Selon Nesher *et al*. [2], aucune documentation clinique ni microbiologique n´est retrouvée dans 40 à 45% des cas (FOI) versus 28% dans notre série. Vingt à 25% des patients ont des sites d'infection identifiables cliniquement mais ont des cultures négatives (FCD), en comparaison à un taux de 54% dans notre étude. Des infections documentées sur le plan microbiologique (FMD) sont retrouvées dans 20 à 25% des cas versus 18% dans notre série. Il est donc primordial de multiplier les prélèvements bactériologiques et particulièrement les hémocultures dans les NF afin d´augmenter les possibilités d´isolement de germes.

Plusieurs schémas thérapeutiques de la NF ont été proposés dans la littérature. Le délai d´administration des antibiotiques est important à respecter. En effet, chaque heure dans le retard de prescription des antibiotiques augmente la mortalité à J28 de 18%. La limite des six heures a été fixée pour le délai d´initiation d´une antibiothérapie empirique, délai au-delà duquel, le risque infectieux est majoré [8]. Nous retenons dans notre série que 62% des épisodes de NF traités par une bithérapie parentérale (C3G+Aminoside) ont bien évolué selon la définition de l´IHS (*Immunocompromised Host Society*) [12]. Toutefois, cette conduite nécessite une hospitalisation et risque d´être contraignante du fait de la durée d´hospitalisation, des couts et du retentissement sur la qualité de vie des patients. C´est pourquoi, l´American *Society of Clinical Oncology* (ASCO) en 2012 puis en 2017 a préconisé une gestion ambulatoire des malades à faible risque par une antibiothérapie orale [13].

Notre étude statistique n´a pas mis en évidence vue la petite taille de l´échantillon, de corrélation significative entre les données cliniques, para-cliniques et la durée de l´aplasie. Toutefois, dans la littérature, différents facteurs pronostiques pouvant avoir un impact tant sur la durée d´hospitalisation et la sévérité de l´épisode d´aplasie que sur la mortalité ont été rapportés [14]: les paramètres hématologiques, la durée de la neutropénie, le type de chimiothérapie, l´âge, le statut de la maladie, le nombre d´épisodes de NF antérieur, la présence d´une infection intercurrente, d´une mucite, la malnutrition, l´atteinte respiratoire, l´isolement d´un germe à la bactériologie et le délai d´administration des antibiotiques. Des études plus larges doivent être menées afin de rechercher une corrélation entre les facteurs cliniques, biologiques avec la durée et la sévérité de la NF.

## Conclusion

Nos résultats concordent avec les données de la littérature concernant la documentation microbiologique, l´épidémiologie bactérienne dominée par les CGP et le taux de mortalité imputée à l´infection. Nos recommandations thérapeutiques ont été efficaces dans 62% des épisodes selon la définition de l´IHS. Au vu de ces résultats, notre choix d´utiliser une antibiothérapie empirique parentérale de 1^ère^ intention associant une C3G à un aminoside parait donc licite permettant ainsi de limiter l´émergence de germes résistants. Toutefois, dans l´avenir, on pourrait envisager après identification des facteurs prédictifs d´évolution défavorable ou de sévérité, une antibiothérapie par voie orale chez les patients à faible risque infectieux.

### Etat des connaissances sur le sujet

- La neutropénie fébrile chimio-induite est fréquente chez l´enfant;
- Les protocoles thérapeutiques diffèrent d´un pays à un autre et d´un service à un autre;
- Il est important d'avoir une stratégie thérapeutique bien codifiée dans les services d'oncologie pédiatrique, adaptée à la flore bactérienne du service et des patients.

### Contribution de notre étude à la connaissance

- Une antibiothérapie probabiliste à base de céphalosporines de 3^e^ génération associées à un aminoside est efficace dans 2/3 des cas des tumeurs solides;
- L'escalade thérapeutique réalisée dans le 1/3 des cas restants permet une évolution favorable dans la plupart des cas;
- La multiplicité des bilans bactériologiques est impérative pour l'adaptation de l'antibiothérapie probabiliste.
